# Invasive Squamous Cell Carcinoma of the Scalp

**Published:** 2015-07-20

**Authors:** Vasanth S. Kotamarti, Adam M. Feintisch, Frank Ciminello

**Affiliations:** Rutgers New Jersey Medical School, Newark

**Keywords:** squamous cell carcinoma, malignancy, dura, scalp, reconstruction

## DESCRIPTION

A 71-year-old man presented with recurrent invasive squamous cell carcinoma (SCC) of the right scalp.

## QUESTIONS

1. **What are the etiology and presentation of squamous cell carcinoma of the scalp?**
2. **What is the significance of depth of invasion in squamous cell carcinoma of the scalp?**
3. **How does the anatomy of the scalp make repair challenging?**
4. **What surgical options are available for reconstruction of scalp defects?**

## DISCUSSION

Risk factors for SCC of the scalp include long-term ultraviolet (UV) or ionizing radiation exposure, arsenic or repeated contact with polycyclic hydrocarbons, and chronic lesions.^1^ The most common etiology is UVB irradiation. Caucasian males with blue eyes, fair hair, and/or alopecia are at greatest risk, particularly those of lower Fitzpatrick skin types. In addition, immunosuppressed patients are susceptible to actinic-induced malignancy and metastasis.^2^ The presentation of face and scalp SCC is often a chronic, nonhealing wound due to a tendency toward ulceration and necrosis.^1^

Depth of invasion is a significant prognostic indicator. In one retrospective study, dural involvement reduced 3-year survival from 83% to 22%. Eisen et al^3^ observed that survival rates did not improve after surgical management of patients' dural extension. Advanced scalp cancer may often be considered inoperable when dura or brain parenchyma is involved. Involvement of a neurosurgeon is necessary to determine safe and adequate resection margins without causing unacceptable impairment.^2^

Several challenges exist when planning scalp reconstruction, particularly hairline cosmesis. Considering the highly visible location and lack of comparable donor site, reconstruction of the hair-bearing scalp may cause significant deformity. Other complicating factors include defect size, previous incisions, and neoadjuvant therapy. High-tension closure and liberal electrocautery may cause follicular destruction and alopecia. Skin inelasticity overlying the galea often precludes primary closure, and significant subgaleal undermining, with or without galeal scoring, may be necessary. The scalp's natural convexity further limits mobility; however, it is well suited for curvilinear incisions used in rotation flaps.^4^

A multidisciplinary approach including a neurosurgeon and a plastic surgeon is oftentimes necessary to ensure safe tumor extirpation and adequate reconstruction. Considerations during reconstruction include contour restoration, hairline maintenance, defect coverage, and return of soft-tissue bulk. In addition, the reconstruction should withstand future shear forces, heal quickly to allow adjuvant treatments, and withstand radiation or trauma.^5^ In patients with extensive malignancies, patient survival takes precedence while function and aesthetics are typically secondary.^6^ Patients requiring radiation therapy present additional concerns. Skin grafting in such cases provides insufficient durability, and staged procedures delay adjuvant therapy. Age provides additional concern, particularly in deep, large defects not amenable to primary or local flap closure; elderly patients often have poor surrounding tissue quality.^7^ Options such as regional or free flaps may be optimal, as they supply healthy tissue with their concomitant blood supply and provide a robust environment for healing, particularly in the event of radiotherapy.

After local options prove inadequate and/or have been exhausted, few options remain, particularly for large defects. Such options include trapezius, anterolateral thigh, and latissimus flaps. Providing ample soft-tissue coverage, the latissimus dorsi flap is a workhorse for scalp reconstruction. It can be elevated as either a regional flap or a free flap, in which case it is often anastomosed to the superficial temporal vessels. In addition, it can be elevated as a pure muscle or musculocutaneous flap. Loss of hair-bearing skin, color, and contour mismatch are concerns when local scalp rearrangement is not possible; however, excessive bulk decreases as the muscle atrophies.^4^

Our patient presented with a history of a recurrent, large, fungating, invasive SCC of the scalp despite previous local resection. Current computed tomographic scan demonstrated an 8 × 8-cm lesion with bicortical calvarial involvement. Tumor resection was accomplished with circumferential 2-cm margins leaving a 12.5 × 12-cm defect. Because of bicortical involvement, craniectomy was performed, revealing dural invasion necessitating excision. Reconstruction included both a pericranial flap and a near-total scalp flap based on the contralateral superficial temporal and terminal ophthalmic vessels. To date, the patient remains tumor-free with stable scalp coverage. He continues to undergo surveillance.

## Figures and Tables

**Figure 1:**
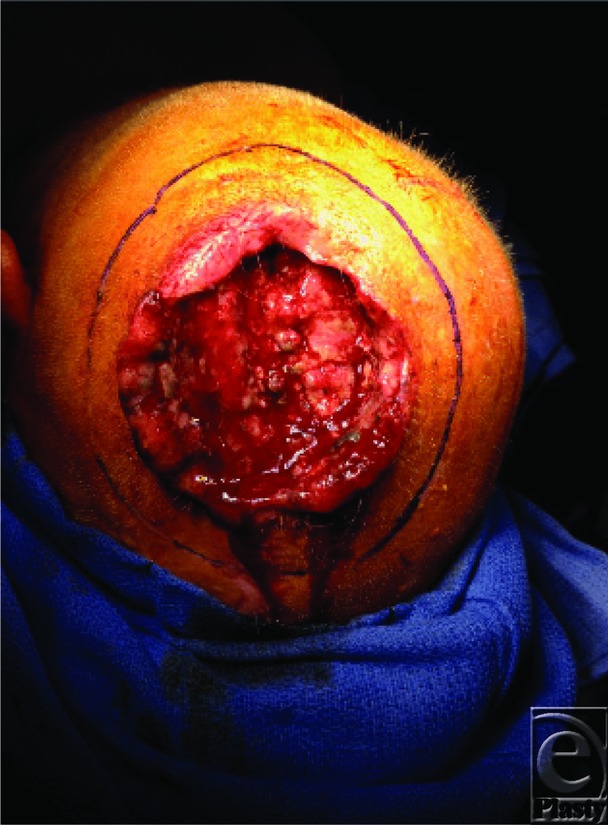
Preoperative photograph of the patient with recurrent, fungating, invasive squamous carcinoma of the scalp. Because of a tendency toward ulceration and necrosis, the presentation was that of a large, nonhealing wound as opposed to a solid tumor.

**Figure 2:**
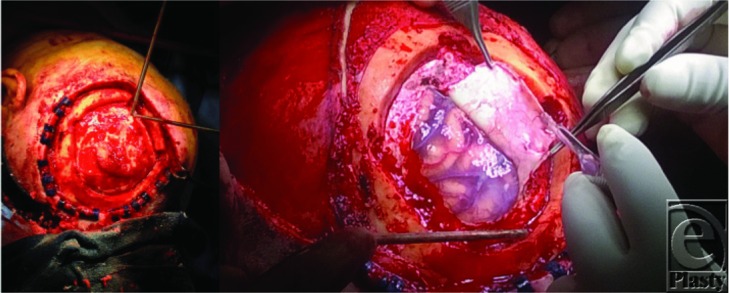
Intraoperative photograph showing craniectomy and excision down to dura. Clips placed along skin edge for hemostasis.

**Figure 3:**
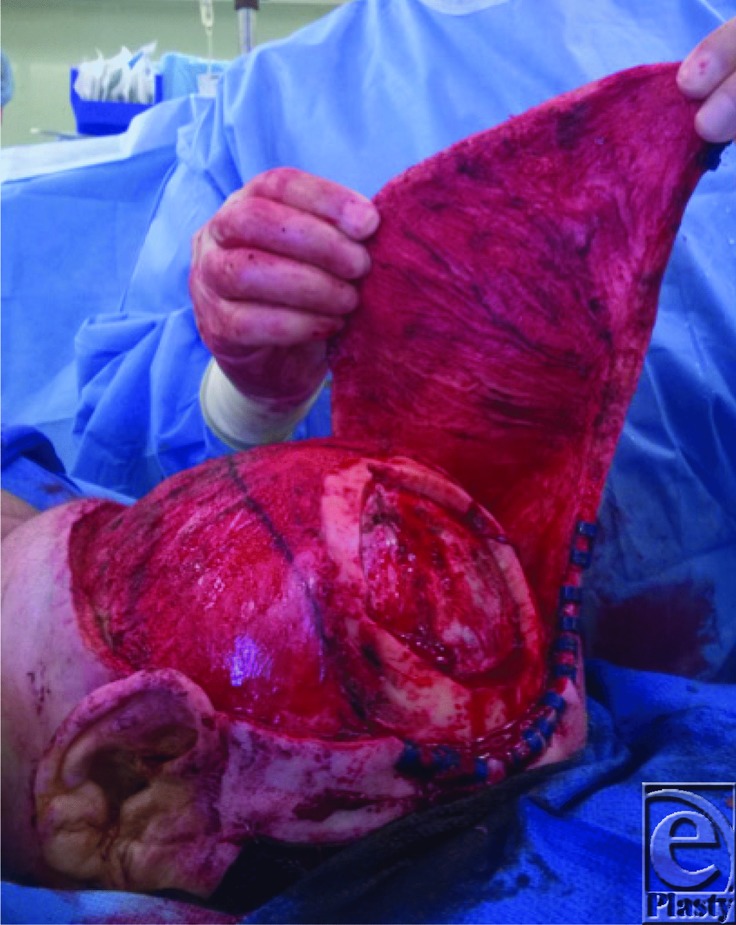
Elevation of scalp flap based off of the contralateral superficial temporal and terminal ophthalmic vessels. A pericranial flap was also accomplished for an additional layer of coverage.

**Figure 4:**
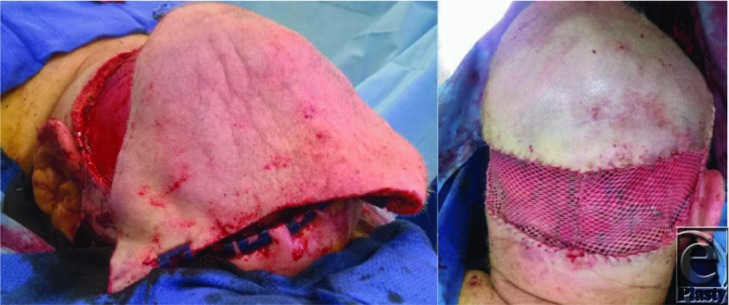
*Left*: Scalp flap arc of rotation showing complete defect coverage. *Right*: Distal aspect of flap trimmed and flap inset. A split-thickness skin graft was meshed and placed over the scalp donor site.
